# Duration of SARS-CoV-2 antigen positivity in hemodialysis patients

**DOI:** 10.1080/0886022X.2021.2013887

**Published:** 2022-01-30

**Authors:** Jun Matsumoto, Yosuke Saka, Tetsushi Mimura, Tomohiko Naruse

**Affiliations:** Department of Nephrology, Kasugai Municipal Hospital, Kasugai City 486-8510, Japan; Department of Nephrology, Kasugai Municipal Hospital, Kasugai, Japan

Dear Editor,

Coronavirus disease (COVID-19), caused by severe acute respiratory syndrome coronavirus 2 (SARS-CoV-2), is an emerging infectious disease that was first reported in December 2019 in Wuhan, China. Older patients with underlying illnesses, such as diabetes mellitus, hypertension, and cardiovascular disease, tend to become severely ill [1]. Most hemodialysis patients have multiple underlying diseases and complications; therefore, SARS-CoV-2 infection in hemodialysis patients has a case fatality rate approaching 20% [2]. Hemodialysis patients also have a very high risk of acquiring SARS-CoV-2 infection compared with the general population [3] because they visit a hemodialysis center several times a week and are in close contact with other patients and caregivers. Therefore, hemodialysis patients are extremely vulnerable to SARS-CoV-2 infection; however, knowledge of their immune response after infection is limited. In this study, we investigated the duration of SARS-CoV-2 antigen positivity in hemodialysis patients with COVID-19.

We retrospectively reviewed the records of 238 consecutive patients hospitalized with COVID-19 at Kasugai Municipal Hospital between 1 February 2020 and 30 June 2021. COVID-19 was defined as a positive SARS-CoV-2 antigen test result. We quantitatively measured the levels of SARS-CoV-2 antigen using the Lumipulse^®^ Presto SARS-CoV-2 Ag (Fujirebio Inc., Tokyo, Japan) assay, performed using a fully automated Lumipulse^®^ L2400 system (Fujirebio Inc., Tokyo, Japan). Patients who were discharged without confirmation of a negative SARS-CoV-2 antigen test result during hospitalization were excluded. Patients were divided into a hemodialysis (HD) group and a non-hemodialysis (non-HD) group. Data on their clinical characteristics, comorbidities, laboratory test results, and treatment type were obtained from their medical records and the clinical characteristics and duration of SARS-CoV-2 antigen positivity in the HD and non-HD groups were compared.

The results were summarized as medians and interquartile ranges (IQRs) or as frequencies and percentages. We performed analysis of covariance to evaluate the duration of antigen positivity after adjustment for parameters with significant differences between the two groups. SPSS Statistics for Windows, version 21 (IBM Corp., Armonk, NY, USA), was used for all statistical analyses. Two-sided *p*-values <.05 were considered to be statistically significant.

The study was approved by Kasugai Municipal Hospital Committee on Human Research (approval number: 445). The requirement for consent was waived because the study was based on a retrospective review of medical records.

A total of 127 patients were included in the analysis, of whom 16 were HD patients and 111 were non-HD patients. Patient baseline characteristics are shown in Table 1. The median age in the HD and non-HD groups was 69.5 years (IQR: 64.8–77.0 years) and 73.0 years (IQR: 58.0–81.0 years), respectively. The proportion of males was 69% and 67%, respectively.

**Table 1.** Baseline characteristics and laboratory findings of patients with COVID-19.

Characteristics	HD (*n* = 16)	non-HD (*n* = 111)	*p* value
Age (yr), median (IQR)	69.5 (64.8−77.0)	73.0 (58.0−81.0)	.733
Male, *n* (%)	11 (69)	74 (67)	1
Body mass index (kg/m^2^) > 35, *n* (%)	3 (19)	7 (6)	.114
Current smoker, *n* (%)	0 (0)	12 (11)	.36
Primary cause of ESKD, *n* (%)			
Diabetic nephropathy	9 (56)	NA	
Hypertension kidney disease	3 (19)	NA	
Glomerulonephritis	3 (19)	NA	
Others	1 (6)	NA	
Coexisting disorder, *n* (%)			
Cardiovascular disease	8 (50)	28 (25)	.071
Diabetes mellitus	10 (63)	24 (22)	.001
Hypertension	13 (81)	47 (42)	.006
Previous medication, *n* (%)			
ACEi/ARB	6 (38)	29 (26)	.375
Presenting symptoms, *n* (%)			
Fever	12 (75)	93 (84)	.477
Cough	13 (81)	30 (27)	<.001
Laboratory findings			
Lactate dehydrogenase (U/L), median (IQR)	253 (221−339)	293 (205−426)	.271
Albumin (mg/dL), median (IQR)	3.2 (2.8−3.5)	3.3 (3.0−3.8)	.143
eGFR (ml/min/1.73m^2^), median (IQR)	NA	65.4 (47.9−83.6)	
C-reactive protein (mg/dL), median (IQR)	7.6 (1.1−9.7)	6.0 (1.8−12.0)	.916
Treatment, *n* (%)			
Remdesivir	3 (19)	28 (25)	.759
Dexamethasone	10 (63)	59 (53)	.595
Outcome			
Mechanical ventilation, *n* (%)	2 (13)	13 (12)	1
Duration of hospitalization (day), median (IQR)	40.0 (24.3−62.0)	19.0 (8.0−34.0)	.003
Death, *n* (%)	2 (13)	12 (11)	.69

IQR: interquartile range; ESKD: end-stage of kidney disease; ACEi: angiotensin-converting enzyme inhibitor; ARB: angiotensin-1 receptor blocker.

The most common primary cause of end-stage kidney disease in the HD group was diabetic nephropathy (56%). Many patients in the HD group had underlying comorbidities with hypertension (81%), diabetes (63%), and cardiovascular disease (50%) being the most common.

Fever was a common symptom in the HD group and the non-HD group (75% and 84%; *p* = .477); however, cough was more frequent in the HD group than in the non-HD group (81% vs 27%; *p* < .001). There were no significant differences between the two groups in albumin, lactate dehydrogenase, and C-reactive protein levels, or the type of treatment provided.

The duration of hospitalization was significantly longer in the HD group than non-HD group (*p* = 0.003), but there were no significant differences between them in the proportions of mechanical ventilation and death.

**Figure 1.**
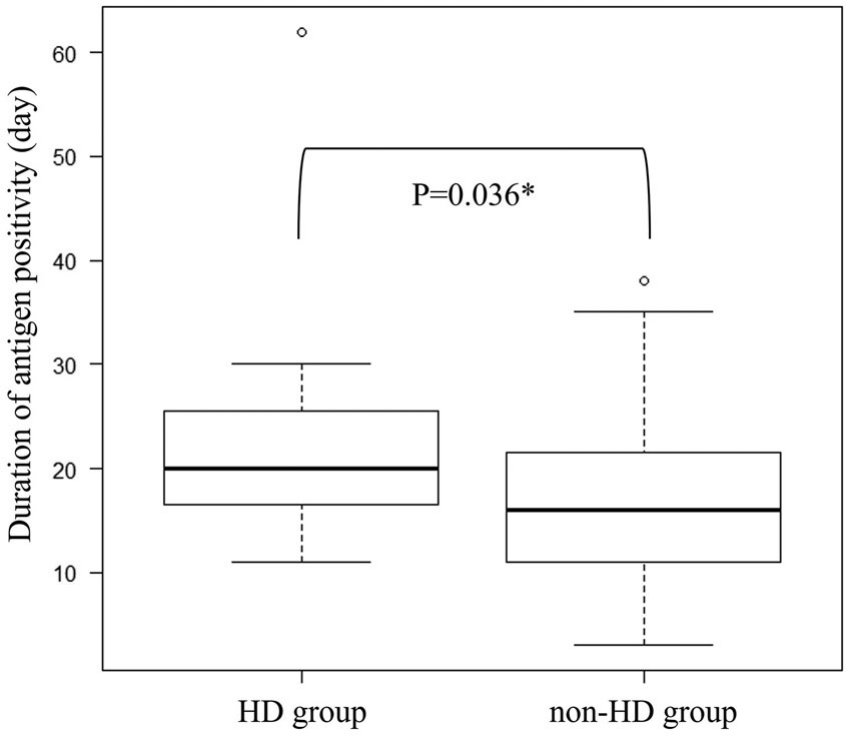
Duration of SARS-CoV-2 antigen positivity in hemodialysis patients and non-hemodialysis patients hospitalized with COVID-19. The duration of SARS-CoV-2 antigen positivity was significantly longer in the HD group than the non-HD group (20 days vs 16 days; *p* = .022). After adjustment for hypertension, diabetes mellitus, and cough, which were the parameters which differed significantly between the groups, the duration of positive SARS-CoV-2 on antigen test remained longer in patients on hemodialysis (*p* = .036). HD: hemodialysis. *Statistically analysis after adjustment

The duration of SARS-CoV-2 antigen positivity was significantly longer in the HD group (median: 20.0 days, IQR: 17.3– 24.8 days) than in the non-HD group (median: 16.0 days, IQR: 11.0 − 21.5 days; *p* = .022) (Figure 1). In the analysis of covariance, the duration of the positive SARS-CoV-2 antigen test result remained significantly longer in patients on hemodialysis (*p* = 0.036) after adjustment for hypertension, diabetes mellitus, and cough, which were parameters with significant differences between the two groups.

To our knowledge, this is the first study to examine the duration of SARS-CoV-2 antigen positivity in hemodialysis patients. The clinical characteristics, laboratory test results, and treatment type of the HD and non-HD groups were similar, yet the duration of antigen positivity was significantly longer in the HD group than in the non-HD group.

Hemodialysis patients have been reported to respond later than healthy age-matched individuals following vaccination with the BNT162b2 (Pfizer-BioNTech) mRNA vaccine [4]. In the case of natural infection, hemodialysis patients do not demonstrate effective clearance of the virus, thus persistence of viral antigens leading to continuous stimulation of immune response with antibody production. The seroconversion rates in hemodialysis patients with COVID-19 have been reported to increase with time [5], but as hemodialysis patients have a deficient immune response [6,7], they may remain SARS-CoV-2 antigen test positive for longer than other patients.

Hemodialysis patients are at a high risk of SARS-CoV-2 infection because of their weakened immunocompetence, need for daily hospital visits, and the possibility of close contact with patients in the dialysis unit [8], which requires significant efforts to expand infection prevention. Currently, SARS-CoV-2 antigen testing is not commonly performed when COVID-19 patients are released from isolation. Considering that the duration of antigen positivity is longer in hemodialysis patients, we suggest that antigen tests be performed in hemodialysis patients before they are released from isolation.
